# A hybrid AI approach for predicting academic performance in RBE students

**DOI:** 10.3389/frai.2025.1651100

**Published:** 2025-10-21

**Authors:** Willy Gonzales, Zindel Cordero, Carlos D. Abanto-Ramírez, Edgar Tito Susanibar Ramírez, Hasnain Iftikhar, Javier Linkolk López-Gonzales

**Affiliations:** 1 Escuela de Posgrado, Universidad Peruana Unión, Lima, Peru; 2 Facultad de Educación, Universidad Nacional José Fausto Sánchez Carrión, Huacho, Peru; 3 Department of Statistics, University of Peshawar, Peshawar, Pakistan

**Keywords:** hybrid AI, artificial intelligence, predicting academic performance, RBE students, statistics

## Abstract

Machine learning has advanced significantly in recent years and is being used in higher education to perform various types of data analysis. While the literature demonstrates the application of machine learning algorithms to predict performance in university education, no such applications are found in EBR, let alone in private institutions of a denominational nature, which presents an opportunity to study prediction in these institutions. To address this gap, this research aims to propose a predictive approach as a decision-support tool for regular basic education, using machine learning techniques. Among the techniques utilized, three machine learning models (Logistic Regression, Support Vector Machine, and Random Forest), along with deep learning models (AlexNet, Gated Recurrent Unit, and Bidirectional Gated Recurrent Unit), were analyzed, as well as ensemble models. Nonetheless, the Ensemble model, which combines deep learning and machine learning techniques, is preferred due to its superior accuracy, precision, and sensitivity performance metrics.

## 1 Introduction

Machine learning (ML) has advanced significantly in recent years and is increasingly applied in higher education (HE) to analyze student performance, predict dropout risk, and support decision-making (Fahd et al., 2022; Mashrur and Nonyelum, 2020; Singh and Kumar, 2020). Educational data mining (EDM) serves as a key methodology for extracting meaningful knowledge and patterns from academic databases, thereby enabling early detection of at-risk students and guiding timely interventions (Sultana and Khan, 2019; Lopez and Ramirez, 2021). A wide variety of ML models–including decision trees (DT), logistic regression (LR), support vector machines (SVM), random forests (RF), and artificial neural networks (ANN)–have been employed to predict academic performance with encouraging results (Ghosh and Sharma, 2022; Hussain and Khan, 2023; Reyes et al., 2025).

Recent studies have also explored deep learning (DL) architectures, such as convolutional neural networks (CNNs) and recurrent neural networks (RNNs), to capture complex temporal and non-linear dependencies in educational datasets (Mi and Yeung, 2019; Ahmed et al., 2022). While these approaches have demonstrated high predictive accuracy in various contexts, including student dropout and performance prediction, most have been developed either with ML or DL models in isolation. In addition, other contributions in AI-driven educational analytics highlight the importance of integrating advanced computational approaches into teaching and learning practices. For example, Tripon (2022) examined how computational thinking skills can be promoted in STEM education through AI-enhanced teaching strategies, demonstrating the growing role of predictive and analytical tools in shaping educational outcomes. Incorporating such perspectives helps to situate our study within the broader landscape of AI applications in education while emphasizing the unique methodological contribution of our ML+DL ensemble approach in the RBE context. Furthermore, recent global research highlights that higher education students are actively forming perceptions about the role of AI tools such as ChatGPT in their learning processes (Ravšelj et al., 2025), offering valuable insights that can inform the design and contextualization of AI-driven predictive models in education. Moreover, applications in Regular Basic Education (RBE) remain limited, and virtually no work has addressed private denominational institutions, leaving a gap in context-specific predictive modeling.

Table 1 summarizes representative studies from the literature, highlighting models used, datasets, and reported accuracies. As shown, ensemble approaches often outperform single models by combining complementary strengths (Menchaca, 2024), yet hybrid ML+DL ensembles remain underexplored in RBE contexts.

**Table 1:** Summary of representative studies on student performance prediction.

**Study**	**Models applied**	**Context**	**Reported accuracy**
Ghosh and Sharma (2022)	SVM, RF	HE, India	SVM: 96.9%, RF: 81.3%
Reyes et al. (2025)	Multinomial LR	HE, Latin America	100% (AUC = 1)
Menchaca (2024)	Ensemble models	HE, Mexico	~90%
Baniata et al. (2024)	GRU	HE, Middle East	99.7%
Fanta and Alemu (2025)	RF	HE, Africa	99% (AUC = 100%)
Mi and Yeung (2019)	DL architectures	HE, Asia	92–96%

In contrast to prior studies that have applied ML or DL models independently (see Chen and Ding, 2023; Mohammadi et al., 2019; Chavez et al., 2023; Adefemi et al., 2025), our work introduces a novel ensemble framework that hybridizes both paradigms. This framework integrates the interpretability and robustness of ML models (e.g., SVM, RF, LR; Smith and Lee, 2017) with the capacity of DL architectures (e.g., AlexNet, GRU, BiGRU) to capture non-linear feature interactions (Brown, 2018; Zhang and Chen, 2021). By explicitly combining these complementary strengths, the ensemble is expected to outperform single-model baselines (cf. Garcia and Alvarez, 2021). Within the RBE institutional context–where datasets often contain both structured attributes and latent dynamic patterns–this hybrid strategy offers a unique methodological advantage that has not been previously investigated (Nguyen and Do, 2019; Castro and Paredes, 2022).

Motivated by the urgent need to strengthen the Peruvian education system, which is ranked 127th out of 137 countries in terms of quality (Pachas, 2020), and aligned with the National Education Project PEN 2036 (Vargas, 2020), this research proposes a predictive framework for private denominational RBE institutions. The approach follows a six-step methodology covering preprocessing, feature selection, normalization, model training, and evaluation. To the best of our knowledge, this is the first study in Peru to employ an ML+DL ensemble for academic performance prediction in RBE, providing an evidence-based decision-support tool for targeted interventions.

The remainder of the paper is structured as follows. Section 2 details the hybrid predictive approach used to evaluate the performance problem of RBE students. Section 3 presents the results and discussion, and Section 4 outlines the conclusions and directions for future research.

## 2 Materials and methods

**Figure 1:**
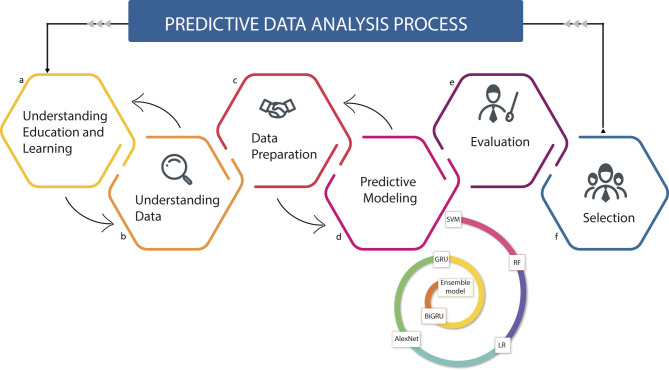
Hybrid predictive approach scheme to evaluate academic performance in students of RBE. It is used to identify key information and significant patterns that allow analyzing the academic performance of students in Regular Basic Education. This approach is developed through six stages that are executed in a sequential and cyclical manner: **(a)** Understanding the educational and learning context, **(b)** Data exploration, **(c)** Data preparation, **(d)** Development of predictive models, **(e)** Evaluation of results, and **(f)** Selection of the best method to measure student performance.

This study uses a predictive approach to evaluate the comparative effectiveness of seven machine learning algorithms in predicting the academic performance of regular basic education students (see Figure 1). This approach allows identifying low-performing students by visualizing the results of predictive models, thus facilitating decision-making by educational institutions. The evaluated algorithms include: Support Vector Machine (SVM), Random Forest (RF), Logistic Regression (LR), AlexNet, Gated Recurrent Unit (GRU), Bidirectional GRU (BiGRU), and an Ensemble model.

### 2.1 Educational and learning comprehension

Academic performance is understood as a way of measuring how the teaching-learning process is developed. It is directly linked to the evaluation of acquired knowledge, so it is necessary to use various instruments that are properly structured and organized (Pacheco et al., 2021). Academic achievement as a complex phenomenon resulting from various personal and social variables, argued as an educational and evaluation element in most countries of the world. This performance is framed within the dynamics of interaction generated daily between students, teachers and the knowledge shared in the educational environment (Santander, 2011). Academic performance is determined by several factors, one of the most relevant of which is the understanding of the student's learning processes, which allows the development of appropriate methodological strategies (Núñez et al., 2022). Academic achievement defined as a complex process that could well be projected as an ascending property of an educational system, and where multiple variables are intertwined, with qualitative and quantitative aspects (Ariza et al., 2018). Other research indicates that academic achievement is linked to individual, psychological, cognitive and intellectual factors, as well as to variables related to educational processes, structural aspects and administrative elements. This perspective provides key concepts for understanding academic achievement as a pedagogical construct of a multicausal, multidimensional and multifactorial nature (Murillo, 2024). The study of academic performance focuses on the grades obtained by students in the first through fifth grades of regular basic education, which represent the outcome of the educational process. In this context, academic performance is considered a fundamental indicator for decision-making at the institutional level, as it enables both the provision of incentives to high-performing students and the implementation of support measures for those with low results.

### 2.2 Understanding data

In this research, characteristics linked to students' academic performance were selected, the most important being the grades obtained from their report cards throughout their time at school. Demographic variables such as gender, location by department and city, and courses completed were also included, as these data provide relevant information about students' personal backgrounds and could have an impact on their academic performance (see Table 2). Meanwhile, the structure and presentation of the data followed the data organization presented in Kord et al. (2025).

**Table 2:** Data description.

**Attribute**	**Data classification**	**Possible values**
Gender	Binary (symmetric)	F-M
Year	Categorical	2019–2021
Association	Categorical	MPS-MPLT-MSOP
Cities	Categorical	Cus-Esp-Pto Mald-Quill-
		Are-Mog-Aza-Jul-Pun
Courses	Categorical	Des Per-Cs Soc-Ed Rel-Ed
		Tra-Ed Fis-Com-Art Cul -
		Ing-Mat-Cie Tec-Ent Vir-
		Apr Aut
Qualification	Float	0–20

### 2.3 Data preparation

#### 2.3.1 Sample selection

The study sample consists of academic data from students at 11 schools belonging to the Adventist Educational Network in southern Peru, in the cities of Arequipa, Moquegua, Puno, Juliaca, Azángaro, Cusco, Espinar, Quillabamba, and Puerto Maldonado. The database includes information collected between 2019 and 2021 (see Table 3). The students in the sample attend regular basic education, ensuring the homogeneity of the group in terms of educational level. No additional exclusion criteria were established, so data from all students at the selected institutions were included in the analysis.

**Table 3:** Detailed description of the dataset variables, including types, ranges, and educational context.

**Number**	**Feature name**	**Type**	**Range**	**Feature description**
1	DPCC	Number	0–20	Personal Development, Citizenship and Civics
2	CS	Number	0–20	Social Sciences
3	ER	Number	0–20	Religious education
4	EPT	Number	0–20	Education for Work
5	EF	Number	0–20	Physical education
6	C	Number	0–20	Communication
7	AC	Number	0–20	Art and Culture
8	I	Number	0–20	English
9	M	Number	0–20	Mathematics
10	CT	Number	0–20	Science and Technology
11	TICs	Number	0–20	It operates in virtual environments generated by ICTs
12	AA	Number	0–20	Manage learning autonomously
13	Grade	Categorical	1st–5th	Grade level
14	Year	Categorical	2019–2021	Year of data
15	Gender	Binary (symmetric)	F – M	Gender of student
16	Place of origin	Categorical	Cities	Place of origin
17	Educational Association	Categorical	Regions	Educational Association

#### 2.3.2 Data collection

The total data set consisted of 155 transcripts from 3,247 Regular Basic Education students with 17 characteristics. These characteristics represent: year of study, place of origin, educational association, academic grade, gender, courses taken, and grades from the first to fifth years (see Table 4). They were classified as qualitative (nominal categorical and ordinal categorical) and quantitative. These variables were processed and analyzed using three machine learning models: Support Vector Machine (SVM), Random Forest (RF), and Logistic Regression (LR); as well as three deep learning models: AlexNet, Gated Recurrent Unit (GRU), and Bidirectional GRU (BiGRU); in addition to an ensemble model. The objective was to determine which of these models offers the best performance in predicting the academic performance of Regular Basic Education students, with a view to contributing to administrative decision-making within educational institutions. The analysis considered 12 subjects: Personal Development, Citizenship and Civics, Social Sciences, Religious Education, Education for Work, Physical Education, Communication, Art and Culture, English, Mathematics, Science and Technology, Develops in virtual environments generated by ICTs and Manages their learning independently. Likewise, five qualitative characteristics were incorporated: gender, place of origin, educational association, year of study and academic degree. Grades are expressed on a vigesimal scale (0 to 20), considering 11 as the minimum passing grade. Likewise, it is important to emphasize that the characteristics of subjects and qualitative characteristics described are similar and coincide across institutions, thus achieving homogeneity in the data.

**Table 4:** Table with processed data from grade transcripts 2019–2021.

**Year**	**Assoc**	**School**	**Grad**	**Gd**	**DPCC**	**CS**	**ED**	**EPT**	**EF**	**C**	**AC**	**1**	**M**	**CT**	**TICs**	**AA**
2019	MSOP	PARDO	Seg A	M	15	13	13	15	16	14	15	17	15	14	15	15
2019	MSOP	PARDO	Seg A	F	18	18	18	18	17	17	19	17	18	16	18	18
2019	MSOP	PARDO	Seg A	F	16	15	18	16	17	17	19	17	18	16	18	18
2019	MSOP	PARDO	Seg A	M	14	12	14	15	15	14	13	12	12	14	13	13

#### 2.3.3 Dataset description and preprocessing

After preprocessing, the final dataset retained 17 features, encompassing academic indicators (such as grades in individual courses and the number of failed courses) along with demographic variables. The predictive task is formulated as a supervised **binary classification** problem. Specifically, the target label classifies students as either **“Pass”** or **“Fail”**, based on their academic results. This formulation allows for a straightforward assessment of academic success and failure, evaluating predictive models more directly aligned with educational decision-making.

Preprocessing was guided by three key criteria: completeness, consistency, and coherence. Records with missing values were imputed using the mean (for numerical attributes) or mode (for categorical attributes). Consistency was ensured by unifying categorical values (e.g., “Female” and “F” both mapped to “F”; “Male” and “M” mapped to “M”), while numerical attributes (e.g., performance scores) were restricted to four decimal places. Coherence was verified by checking for outliers and distributional anomalies. Outlier detection revealed that some exchange students and Theology majors were older than the average student body. Still, these cases were retained since they aligned with the overall trends of the dataset.

To enable machine learning models to operate effectively, quantitative attributes were standardized to a mean of 0 and a standard deviation of 1. Each variable *X*_*i*_ was transformed into its standardized form *Z*_*i*_ using:


$$Z_{i}=\frac{\left(X_{i}-\bar{X}\right)}{S_{n-1}},\;i=1,\ldots ,n,$$

where


$$\bar{X}=\frac{1}{n}\overset{n}{\underset{i=1}{\sum }}X_{i},\;S_{n-1}=\sqrt{\frac{1}{n-1}\overset{n}{\underset{i=1}{\sum }}{\left(X_{i}-\bar{X}\right)}^{2}}.$$

#### 2.3.4 Feature selection

To reduce dimensionality and enhance interpretability, a two-stage feature selection procedure was adopted:

1. **Correlation analysis**. Pairwise correlations between predictors and the target variable were computed. Features with negligible association to the outcome or those exhibiting strong multicollinearity (Pearson's *r*>0.85) were flagged for removal.
2. **Recursive Feature Elimination (RFE)**. Using logistic regression as a base estimator, RFE iteratively eliminated the least essential predictors based on coefficient weights. Cross-validation determined the optimal subset of features by maximizing predictive accuracy on the validation set.

This hybrid strategy combines the statistical efficiency of correlation analysis with the model-based refinement of RFE. As a result, the feature set was reduced from 22 to 12 attributes without loss of predictive power. In fact, models trained on the reduced set achieved slightly higher accuracy, F1-score, and AUC, highlighting both the computational efficiency and predictive robustness of the streamlined dataset.

### 2.4 Predictive modeling

This section details how the predictive models in this work were used to predict student performance. Hence, three machine-learning models, such as the Logistic regression model, Support Vector Machine model, and Random forest model; three deep learning models, including the Alexnet, the Gated Recurrent Unit, and Bidirectional Gated Recurrent Unit; and their proposed ensemble model. The details of each model are given below.

#### 2.4.1 Logistic regression

The linear regression (LGR) is the basic method of binary classification, which estimates the probability of observation belonging to a particular category. The sigmoid function converts linear combinations of features into probability values. The LGR model requires careful parameter tweaking and is best suited for binary classification problems. However, while using this strategy, it is critical to keep certain assumptions in mind, such as the assumption of feature independence. The LGR model is mathematically represented as follows: Let X represent an instance's feature vector, and y represent the binary class label (0 or 1). Given X, the logistic regression (LGR) model computes the probability that y = 1. This probability is denoted as:


$$\begin{array}{l} P\left(y=1/X\right)=\frac{1}{1+e^{-\left(\alpha_{0}+\alpha_{1}X_{1}+\alpha_{2}X_{2}+\ldots +\alpha_{P}X_{P}\right)}} \end{array}$$ (1)

where *P*(*y* = 1/*X*) represents the probability that the class label *y* is one given the feature vector *X*, while *e* means the base of the natural logarithm (~2.71828), and α_0_, α_1_, α_2_, …, α_*P*_ are the coefficients (parameters) of the model to be learned during training. *X*_1_, *X*_2_, …, *X*_*P*_ are the features of the instance X. The sigmoid function $\frac{1}{1+e^{-z}}$ maps the linear combination of features α_0_+α_1_*X*_1_+α_2_*X*_2_+…+α_*P*_*X*_*P*_ generates a value between 0 and 1, which indicates the probability that the observation is assigned to class 1. The model assigns the example to the class with the higher probability. Despite the effectiveness of logistic regression in binary classification, it is crucial to verify the validity of its assumptions, such as the assumption of linearity and feature independence, as these can impact its performance in real-world applications. Proper parameter tuning is also crucial for achieving optimal results with logistic regression.

#### 2.4.2 Support vector machine

Support Vector Machine (SVM) model is one of the most commonly used ML algorithms to identify a hyperplane in N-dimensional space that classifies the data points. It helps find a plane that maximizes the margin. The diversity of N-dimensional space is based on the number of features; two features can be smoothly compared. However, it is more complex in the case of several features in classification. Maximizing the margin leads to a more accurate prediction. The margin refers to the distance between the decision hyperplane and the nearest instances, which is the number of that class. Most of the time, data is not linearly separable; in that case, SVM uses various types of functions called kernels to transform the data into the desired shape through input vectors. This study uses a linear kernel function. A linear kernel is one of the most straightforward functions for transforming data into the desired shape. These functions return the inner product between two suitable feature points in a dimensional space.


$$\begin{array}{l} K\left(y_{i},y_{j}\right)=\left(y_{i}.y_{j+1}\right) \end{array}$$ (2)

#### 2.4.3 Random forest model

The ensemble learning technique known as Random Forest (RF) combines the predictive strength of many decision trees with the addition of randomization to reduce overfitting. RF produces multiple decision trees and uses bootstrapping to train each tree using a different data part. The final ranking is determined by combining the results from the individual tree, which can be achieved by majority voting for classification tasks or by averaging for regression tasks. Mathematically, let's represent a Random Forest ensemble as a set of decision trees (*T*_1_, *T*_2_, …, *T*_*n*_), where each tree *T*_*i*_ is trained on a different bootstrapped sample from the original training dataset. The classification for a new sample *X*_new_ using the Random Forest ensemble can be expressed as follows:

For classification


$$\begin{array}{l} {ŷ}_{\text{RF}}\left(X_{\text{new}}\right)=\text{MajorityVote}\left(T_{1}\left(X_{\text{new}}\right),T_{2}\left(X_{\text{new}}\right),\ldots ,T_{n}\left(X_{\text{new}}\right)\right) \end{array}$$ (3)

For regression


$$\begin{array}{l} {ŷ}_{\text{RF}}\left(X_{\text{new}}\right)=\frac{1}{n}\overset{n}{\underset{i=1}{\sum }}T_{i}\left(X_{\text{new}}\right) \end{array}$$ (4)

where: ŷ_RF_(*X*_new_) represents the final prediction or classification for the new sample *X*_new_ using the Random Forest ensemble. *T*_*i*_(*X*_new)_ represents the prediction or classification made by the *i*^*t*^*h* decision tree in the ensemble for the new sample. Majority Vote (.) calculates the majority vote among the individual tree predictions for classification tasks, and *s* is the total number of decision trees in the RF ensemble. RF is powerful. After all, it may average or combine the outputs of several trees, each trained on a separate subset of the data, because it can decrease overfitting. The model's resilience and capacity for generalization are improved by this ensemble technique, making it a popular option for various machine-learning applications.

#### 2.4.4 The AlexNet model

One of the significant developments in computer vision and deep learning, AlexNet, is a large-scale convolutional neural network (CNN) architecture that has significantly increased picture classification precision in Image Network datasets. Three entirely interconnected layers, one final softmax layer for classification, and five convolutional layers make up an AlexNet architecture. A number of new methods have also been introduced, such as dropout regularization to prevent over-adjustment, data improvement by picture reflection and cropping, and the use of rectified linear units (ReLUs) as activation functions. AlexNet's primary contribution was paving the way for further developments in the field of image identification by demonstrating the efficacy of deep neural networks for such tasks. Currently, a lot of the most sophisticated CNN architectures are built on top of AlexNet's foundations and keep pushing the limits of computer vision applications like image recognition.

#### 2.4.5 The gated recurrent unit model

The RNN design known as the Gated Recurrent Unit (GRU) solves the fading gradient issue and makes it possible to identify persistent dependencies in the order of data. It is presented as a more straightforward and effective design substitute for conventional long-term memory units (LSTMs). GRU units are made up of update and reset gates to regulate the information flow within the network. The updated door determines what exactly should be added to the new input and what should be kept from the prior hidden state. By pressing the reset button, the network may calculate the number of hidden states from the past that are important to the input at hand. GRU can record both short- and long-term dependencies with these gates by selectively updating and resetting its hidden state in response to the input sequence. GRU's streamlined architecture, which requires fewer parameters and expedites training, is one of its advantages over LSTM. This is especially helpful when working with huge data sets or constrained computational resources. Additionally, GRU and LSTM demonstrated comparable performance in a number of tasks, including machine translation, sentiment analysis, language modeling, and language recognition. Because GRU technology can manage both short- and long-term dependencies, it has shown effectiveness in modeling sequence data. It is extensively utilized in many different domains, such as sequence data production, time series analysis, and natural language processing. To increase efficiency and accuracy, scientists and industry professionals are still investigating and fine-tuning GRU-based models and their modifications and combinations with other methods.

#### 2.4.6 The bidirectional gated recurrent unit model

A general-purpose deep learning architecture called the Bidirectional Gated Recurrent Unit (BiGRU) is utilized for sequential data modeling, including time series, text, and speech data. It is a development of the conventional GRU design that adds two-way processing to let networks remember past, present, and future input patterns. Two contemporaneous GRU layers comprise the architecture; one processes the order of inputs forward and the other backward. The outputs from these two layers are concatenated when going through dense layers for regression and classification. For applications involving natural language processing, including machine translation, sentiment analysis, and named entity recognition, BiGRU is especially helpful when information about previous and upcoming input sequences is available. BiGRU is frequently utilized as a benchmark model to assess more intricate designs against, as it has demonstrated state-of-the-art performance for an extensive array of workloads. All things considered, sequential data modeling has been using BiGRU, a strong and adaptable deep-learning method, more and more in recent years. Time sequence data, speech, text, and other sequential data types are frequently processed using RNNs, a deep learning model. RNNs are made to work with variable-length sequences, in contrast to classic neural networks, which accept fixed inputs and generate fixed outputs. This is accomplished by adding loops to the network that allow information to endure over time. Because of this, they are especially good at processing stimuli that are sequential or have a temporal component.

#### 2.4.7 The proposed ensemble model

Ensemble learning is an approach for improving model accuracy and efficacy. It is a powerful meta-learning strategy that combines weak and strong learners to boost the effectiveness of the weak learner. This article uses the ensemble approach to increase the accuracy of several models for BC illness prediction. Combining several models tries to improve performance over individual models. This work creates an ensemble using six models: LGR, SVML, RF, Alexnet, GRU, and BiGRU. The ensemble model is built using a weighting approach. The weighted average ensemble approach allows several models to contribute to a forecast based on their confidence level or anticipated performance. Each member's contribution to the final forecast is weighed against the model's performance in a weighted ensemble. The model weights are tiny positive numbers totaling one, reflecting each model's degree of trust or predicted performance. This work assigns the Ensemble model weights: LGR: 0.1511, SVM: 0.2151, RF: 0.1179, Alexnet: 0.1912, GRU: 0.1329, and BiGRU: 0.1818.

### 2.5 Evaluation

The proposed intelligence hybrid system has been evaluated by six different performance metrics (accuracy, sensitivity, specificity, F1 score, Brier score, and error rate), an equal prediction test statistical test (the Diabold-Marino test), and a visual analysis (bar plot, line plot, and level plot; Iftikhar et al., 2023; Cuba et al., 2024; Gonzales et al., 2024; Alshanbari et al., 2023). The specifications are as follows:


$$\begin{array}{l} \text{Accuracy}=\frac{\text{TP+TN}}{\text{TP+FP+TN+FN}}, \end{array}$$ (5)

The following formulas show that TP is a true positive number, TN is a false negative number, FP is a false positive number, and FN is a false negative number.


$$\begin{array}{lll} \text{Sensitivity} & = & \frac{\text{TP}}{\text{TP}+\text{FN}}. \end{array}$$ (6)

$$\begin{array}{lll} \text{Specificity} & = & \frac{\text{TN}}{\text{TN}+\text{FP}} \end{array}$$ (7)

$$\begin{array}{lll} F1-Score & = & 2\times \frac{\text{Precision}\times \text{Recall}}{\text{Precision}+\text{Recall}}, \end{array}$$ (8)

The F1 score ranges from zero to one. A score near to one implies more incredible model performance, whereas a value close to zero indicates poor performance.


$$\begin{array}{l} \text{Brier Score}=\frac{1}{n}\overset{n}{\underset{i=1}{\sum }}{\left(y_{i}-\hat{y_{i}}\right)}^{2}, \end{array}$$ (9)

where *n* is the number of instances, *y*_*i*_ is the observed binary output, and $\hat{y_{i}}$ is the predicted binary output for instance *i*. Brier Score ranges between 0 and 1, where a value closer to zero indicates better performance, while a value close to 1 shows poor performance of the predictive models.


$$\begin{array}{l} \text{Error}=\left(y_{i}-\hat{y_{i}}\right), \end{array}$$ (10)

where *y*_*i*_ represents actual value and $\hat{y_{i}}$ denotes the predicted value in specific data points.

Furthermore, to the performance metrics, the Diebold-Mariano (DM) test has been performed to examine the significance of the variations in the prediction performance of the predictive models (Diebold and Mariano, 2002). This equal prediction test is commonly used to compare predictions from various models (Qureshi et al., 2024; Iftikhar et al., 2024b; Qureshi et al., 2025; Iftikhar et al., 2024a). The DM statistic may be calculated using the following equation:


$${\text{DM}}_{s}\;=\;\frac{\bar{y}}{\sqrt{\text{Var}(\bar{y})}},$$ (11)

where,


$$\bar{y}\;=\;\frac{1}{\text{H}}\sum_{\text{h=1}}^{\text{H}}{\text{y}}_{\text{h}}\text{, }{\text{y}}_{\text{h}}\;=\;{\left(c_{\text{h}}\;-\;{\tilde{c}}_{1\text{h}}\right)}^{2}\;-\;{\left(c_{\text{h}}\;-\;{\tilde{c}}_{2\text{h}}\right)}^{2},$$ (12)

$$\text{Var}(\bar{y})\;=\;\frac{1}{\text{H}}(2\sum_{j=1}^{\text{h }-\text{ 1}}r_{j}+r_{0}),and\;r_{j}\;=\;\mathrm{cov}({\text{y}}_{\text{h}}\;-\;{\text{y}}_{\text{h }-\text{j}}).$$ (13)

the estimated value of the first predicting model is ${\tilde{\text{c}}}_{1\text{h}}$, whereas the predicted value of the second predicting model at time h is ${\tilde{c}}_{2\text{h}}$.

### 2.6 Selection

The results obtained after applying various algorithms to data from regular basic education students show that the ensemble model performs best in predicting academic performance, standing out for its high precision, accuracy, and sensitivity. This suggests that its implementation could be useful for educational institutions to design more effective policies and strategies aimed at strengthening and supporting students based on their estimated performance.

## 3 Results and discussion

Table 5 provides the performance metrics of various machine learning, deep learning, and ensemble models assessed through a cross-validation method over 500 iterations. It is categorized into three distinct scenarios, each representing a different split of training and testing data: 50%–50% (Scenario 1), 75%–25% (Scenario 2), and 90%–10% (Scenario 3). The models evaluated include Support Vector Machine (SVM), Random Forest (RF), Logistic Regression (LR), AlexNet, Gated Recurrent Unit (GRU), Bidirectional GRU (BiGRU), and an Ensemble model.

**Table 5:** Performance measures: results of the evaluation of the deep learning, machine learning, and ensemble models with a cross-validation approach of 500 executions.

**Models**	**Accuracy**	**Sensitivity**	**Specificity**	**FS**	**BS**	**Error**
**1st scenario (50%, 50%)**						
SVM	0.9055	0.9538	0.9538	0.9599	0.0242	0.0945
RF	0.8826	0.9217	0.9217	0.9281	0.0359	0.1174
LR	0.8854	0.9296	0.9296	0.9388	0.0325	0.1146
Alexnet	0.9089	0.9582	0.9582	0.9641	0.0208	0.0911
GRU	0.8953	0.9518	0.9518	0.9467	0.0258	0.1047
BiGRU	0.8994	0.9522	0.9522	0.9515	0.0272	0.1006
Ensemble	0.9244	0.9715	0.9715	0.9774	0.0187	0.0756
**Models**	**Accuracy**	**Sensitivity**	**Specificity**	**F1 score**	**Brier score**	**Error**
**2nd scenario (75%, 25%)**						
SVM	0.9111	0.9594	0.9594	0.9655	0.0216	0.0889
RF	0.8882	0.9273	0.9273	0.9337	0.0333	0.1118
LR	0.8910	0.9352	0.9352	0.9444	0.0299	0.1090
Alexnet	0.9145	0.9638	0.9638	0.9697	0.0182	0.0855
GRU	0.9009	0.9574	0.9574	0.9523	0.0232	0.0991
BiGRU	0.9050	0.9578	0.9578	0.9571	0.0246	0.0950
Ensemble	0.9244	0.9715	0.9715	0.9774	0.0187	0.0756
**3rd scenario (90%, 10%)**						
SVM	0.9137	0.9637	0.9637	0.9698	0.0196	0.0864
RF	0.8908	0.9316	0.9316	0.9380	0.0313	0.1093
LR	0.8936	0.9395	0.9395	0.9487	0.0279	0.1065
Alexnet	0.9171	0.9681	0.9681	0.9740	0.0162	0.0830
GRU	0.9035	0.9617	0.9617	0.9566	0.0212	0.0966
BiGRU	0.9076	0.9621	0.9621	0.9614	0.0226	0.0925
Ensemble	0.9244	0.9715	0.9715	0.9774	0.0187	0.0756

Each model is assessed based on metrics such as Accuracy, Sensitivity, Specificity, F1 Score, Brier Score (BS), and Error Rate. In all three scenarios, the Ensemble model consistently delivers the highest scores in terms of accuracy, sensitivity, specificity, and F1 score, while also achieving the lowest error rates and Brier scores. This indicates that integrating multiple models leads to enhanced performance and robustness.

When analyzing individual models, deep learning-based frameworks like AlexNet, GRU, and BiGRU generally outperform conventional machine learning models such as SVM, RF, and LR in most instances. AlexNet and BiGRU show strong sensitivity and specificity values, highlighting their effectiveness in accurately identifying both positive and negative instances. Among the machine learning models, SVM ranks highest, followed closely by LR and RF.

As the proportion of training data increases across the scenarios (from 50% in Scenario 1 to 90% in Scenario 3), the accuracy, sensitivity, specificity, and F1 score tend to rise, while the error rate and Brier score tend to decrease. This illustrates that a larger training dataset aids in enhancing model generalization and performance. In summary, the findings highlight the advantages of deep learning techniques compared to traditional machine learning models in this classification challenge. Furthermore, the ensemble model emerges as the top performer across all evaluated metrics, making it the most dependable option for this particular issue.

On the other hand, after evaluating the model performance with accuracy metrics (accuracy, sensitivity, specificity, F1 score, Brier score, and error), the same prediction statistical tests (DM tests) were used to explore the significance of different prediction abilities between models. Table 6 shows the DM test of each model pair to determine the outcome quality (mean performance measurement). The results of the DM test (*p*-values) are shown in Table 6. For clarity, the DM test examines whether the forecasting errors of two models are statistically different. A *p*-value below 0.05 indicates that the difference in predictive performance between two models is statistically significant at the 5% level, while higher *p*-values suggest that the models perform similarly. In this study, the table confirms that the ensemble model consistently achieves statistically significant improvements compared to the other six prediction models across all data-splitting scenarios.

**Table 6:** All predictive models (*p*-values) of DM test results are considered in all training and test dataset scenarios.

**Model**	**SVML**	**RF**	**LGR**	**Alexnet**	**GRU**	**BiGRU**	**Ensemble**
**1st scenario (50%, 50%)**						
SVML	0.00	0.02	0.01	0.99	0.06	0.05	0.99
RF	0.98	0.00	0.98	0.98	0.98	0.98	0.99
LGR	0.99	0.03	0.00	0.99	0.97	0.98	0.99
Alexnet	0.01	0.02	0.01	0.00	0.03	0.02	0.99
GRU	0.94	0.02	0.03	0.97	0.00	0.93	0.98
BiGRU	0.95	0.02	0.02	0.98	0.07	0.00	0.98
Ensemble	0.01	0.01	0.01	0.01	0.02	0.02	0.00
**2st scenario (75%, 25%)**						
SVML	0.00	0.02	0.01	0.99	0.06	0.05	0.99
RF	0.99	0.00	0.98	0.99	0.98	0.98	0.99
LGR	0.99	0.02	0.00	0.99	0.97	0.98	0.99
Alexnet	0.01	0.02	0.01	0.00	0.03	0.02	0.99
GRU	0.94	0.02	0.03	0.97	0.00	0.93	0.98
BiGRU	0.95	0.02	0.02	0.98	0.07	0.00	0.99
Ensemble	0.01	0.01	0.01	0.01	0.02	0.01	0.00
**3st scenario (90%, 10%)**						
SVML	0.00	0.01	0.01	0.99	0.06	0.05	0.99
RF	0.99	0.00	0.98	0.99	0.98	0.98	0.99
LGR	0.99	0.02	0.00	0.99	0.97	0.98	0.99
Alexnet	0.01	0.01	0.01	0.00	0.03	0.02	0.99
GRU	0.94	0.02	0.03	0.97	0.00	0.93	0.98
BiGRU	0.95	0.02	0.02	0.98	0.07	0.00	0.99
Ensemble	0.01	0.01	0.01	0.01	0.02	0.01	0.00

**Figure 2:**
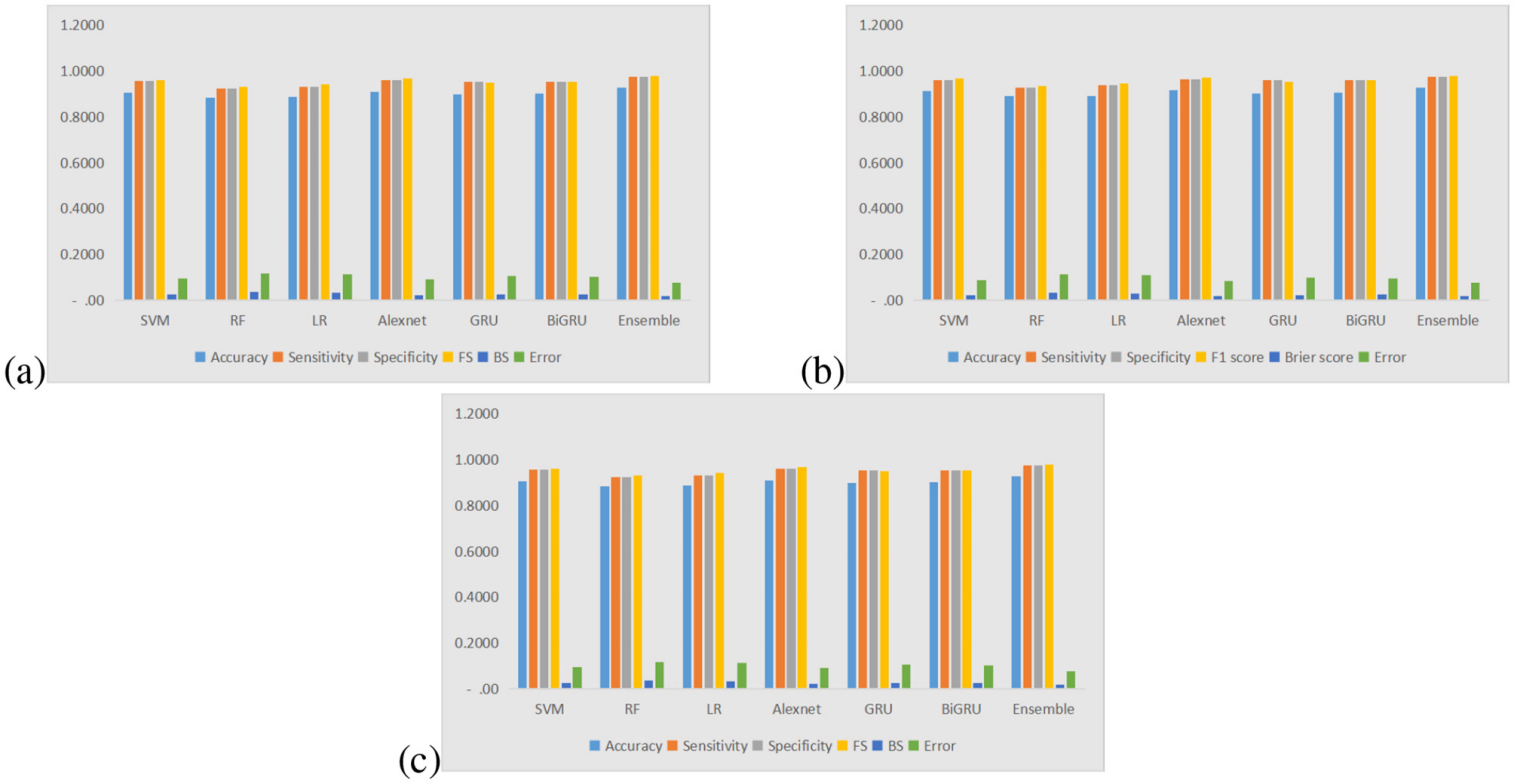
Performance measures bar plots for all predictive models for all three scenarios: **(a)** 50% training and 50% testing, **(b)** 75% training and 25% testing, **(c)** 90% training and 10% testing.

Figure 2 illustrate the performance metrics of various predictive models evaluated under three distinct scenarios: (a) 50% training data and 50% testing data, (b) 75% training data and 25% testing data and (c) 90% training data and 10% testing data. The models considered include Support Vector Machine (SVM), Random Forest (RF), Logistic Regression (LR), AlexNet, Gated Recurrent Unit (GRU), Bidirectional GRU (BiGRU), and an Ensemble model. The metrics employed for evaluation consist of Accuracy, Sensitivity, Specificity, F1 Score (FS), Brier Score (BS), and Error. Throughout all scenarios, the models demonstrate consistently high levels of accuracy, sensitivity, specificity, and F1 scores, indicating their effectiveness in classification tasks. The Ensemble model consistently achieves the best results, exhibiting minimal error and advanced classification abilities.

- In scenario **(a) 50% training–50% testing**, the models show somewhat lower accuracy relative to scenarios with a greater amount of training data. This indicates that a smaller training dataset causes a slight decrease in the models' generalization capabilities.
- In scenario **(b) 75% training–25% testing**, the performance across all models improves, with higher accuracy and decreased error. This underscores the notion that more training data positively influences model performance.
- In scenario **(c) 90% training–10% testing**, the models achieve optimal performance, as evidenced by the highest accuracy and the lowest error rates. The Ensemble model particularly distinguishes itself as the most dependable model, demonstrating strong generalization capabilities.
- **SVM, RF, and LR**: these traditional machine learning models display competitive results but tend to fall short compared to deep learning models (AlexNet, GRU, and BiGRU) regarding sensitivity and specificity.
- **AlexNet, GRU, and BiGRU**: these deep learning models illustrate enhanced classification capabilities, particularly in sensitivity and F1 score. Their effectiveness in learning intricate patterns within data contributes to their outstanding performance.
- **Ensemble model**: this model consistently surpasses all other models, suggesting that integrating multiple classifiers enhances predictive accuracy and robustness.

The Brier score and error metrics decrease with an increase in the training data proportion, signifying that more training data results in better-calibrated predictions and fewer misclassification incidents. The lowest error rates are recorded in the **90% training–10% testing** scenario, reinforcing the advantages of augmented training data. This analysis reveals that enhancing the amount of training data substantially boosts model performance across all evaluated metrics. Deep learning models (AlexNet, GRU, BiGRU) and the Ensemble model consistently outperform conventional machine learning methods. Notably, the Ensemble model exhibits superior classification accuracy, sensitivity, and specificity, positioning it as the most trustworthy predictive model in all tested scenarios. These insights highlight the value of employing ensemble and deep learning approaches for critical classification tasks, particularly when ample training data is available.

## 4 Conclusions

The findings allow us to conclude that the chosen deep learning, machine learning, and ensemble methods demonstrated a strong predictive ability, primarily due to the linear correlation between academic characteristics and student outcomes. Among the techniques utilized, three machine learning models (Logistic Regression, Support Vector Machine, and Random Forest), along with deep learning models (AlexNet, Gated Recurrent Unit, and Bidirectional Gated Recurrent Unit), were analyzed, as well as ensemble models. Nonetheless, the Ensemble model, which combines deep learning and machine learning techniques, is preferred due to its superior accuracy, precision, and sensitivity performance metrics. The critical factors for assessing a student's academic performance at the Peruvian university include the number of Failed Courses and the grades received in the first 2 years, as these are crucial determinants for the student's academic success. To ensure that a student maintains good academic performance in subsequent years, proactive measures should be taken in alignment with the model's predictions during the initial two years. Furthermore, the consistency of the proposed predictive system was validated by dividing the entire dataset into three training and testing scenarios [(90%, 10%), (75%, 25%), and (50%, 50%)], conducting a comparative assessment of the models using six performance metrics, graphical analyses, and statistical testing through five hundred simulation runs. According to the evaluation results, the ensemble model consistently outperformed the other models across all three training and testing scenarios.

Despite the promising results, several limitations must be acknowledged. First, the dataset is restricted to 11 schools of the Adventist Educational Network in the macro-south of Peru, which raises questions about external validity. The predictive power of the model may be context-dependent, as socio-economic, cultural, and institutional factors not captured in the dataset could significantly influence academic performance in other settings. Therefore, extrapolation of results beyond this specific institutional and geographical context should be approached with caution. Second, although rigorous data cleaning and verification procedures were performed, reliance on institutional records may still introduce biases or gaps in data collection that could affect the outcomes. Finally, while the models achieve high predictive performance, the absence of explicit interpretability analysis (e.g., SHAP or LIME) limits their immediate practical applicability for educators and decision-makers.

Future research should address these limitations by (i) testing the model on external datasets from diverse institutional and cultural contexts to assess generalizability, (ii) incorporating broader socio-economic and demographic variables to capture a wider range of determinants of student success, (iii) evaluating longitudinal datasets to study dynamic academic trajectories, and (iv) integrating explainability frameworks to provide actionable insights into the role of specific features. By pursuing these directions, subsequent studies can enhance the robustness, fairness, transparency, and practical utility of AI-driven predictive systems in education. Likewise, can also be extended to other scenarios with different datasets (Iftikhar et al., 2024c; Cabello-Torres et al., 2022; Cruz et al., 2020).

## Data Availability

The raw data supporting the conclusions of this article will be made available by the authors, without undue reservation.
